# Integrated Analysis of Remdesivir and Paxlovid in COVID-19 Patients: A Personalized Approach to High-Risk Individuals for Severe Evolution

**DOI:** 10.3390/jcm13226670

**Published:** 2024-11-06

**Authors:** Andreea Fitero, Nicoleta Negrut, Anca Popa, Harrie Toms John, Anca Cristina Ferician, Felicia Manole, Paula Marian

**Affiliations:** 1 Department of Psycho-Neuroscience and Recovery, Faculty of Medicine and Pharmacy, University of Oradea, 410073 Oradea, Romania; andreea_gyori@yahoo.com; 2 Doctoral School of Biomedical Sciences, Faculty of Medicine and Pharmacy, University of Oradea, 410087 Oradea, Romania; manole.felicia@gmail.com; 3 Department of Endocrinology, Emergency Clinical County Hospital of Oradea, 410169 Oradea, Romania; ancapopa@uoradea.ro; 4 Department of Animal Science and Agritourism, Faculty of Environmental Protection, University of Oradea, 410087 Oradea, Romania; 5 Department of Anesthesia and Intensive Care, Emergency Clinical County Hospital of Oradea, 410169 Oradea, Romania; 6 Department of Medical Disciplines, Faculty of Medicine and Pharmacy, University of Oradea, 410073 Oradea, Romania; anca.moza@yahoo.com (A.C.F.); paula.marian85@gmail.com (P.M.); 7 Department of Surgical Disciplines, Faculty of Medicine and Pharmacy, University of Oradea, 410073 Oradea, Romania

**Keywords:** COVID-19, remdesivir, Paxlovid, SARS-CoV-2

## Abstract

**Background/Objectives**: COVID-19 led to a pandemic that has brought misery to millions of people but more so to those with pre-existing conditions. For this infection, several antiviral drugs were employed, including remdesivir (R) and Paxlovid (nirmatrelvir/ritonavir (NR)). **Methods**: The current study compared the effectiveness of remdesivir and Paxlovid treatment for COVID-19 patients with comorbid conditions. Data from a cohort of 151 adult patients with COVID-19 who also had associated comorbidities were used in this study. These patients were treated with antivirals according to local guidelines. The subjects included 78 case-patients assigned to group R and 73 to group NR. **Results**: In group NR, a considerable improvement in oxygen saturation was seen in the first 24 h of treatment (*p* = 0.010), but the levels were significantly higher from the second day of treatment (*p* < 0.001) in group R of patients. At the end of the 5 days of treatment, the oxygen saturation improved statistically significantly compared to the admission day, but only in the R group (95.11 ± 1.80; 91.76 ± 1.80; *p* < 0.001). **Conclusions**: Both drugs can be considered a breakthrough in the current treatment approach to the COVID-19 disease since they provide readily available options that can alleviate the severity of the disease and, hence, the prognosis of patients. That is why their effectiveness relies on the correct administration time and choosing the patient with suitable characteristics regarding the presence of comorbidities and the likelihood of the critical further development of the process.

## 1. Introduction

The novel coronavirus (SARS-CoV-2) pandemic has led to the quick development and application of different therapeutic approaches, which can help minimize severe consequences in infected patients. SARS-CoV-2, the causative agent of COVID-19, is a ribonucleic acid (RNA) virus characterized by high transmissibility and significant impact on global health systems. The rapid progression of the disease towards forms with multisystem involvement, organ failure, and the need for critical intervention, including acute respiratory failure, is recognized as severe progression in COVID-19 patients. Risk factors such as advanced age, comorbidities, and immunosuppressive states facilitate this progression. Its ability to cause severe respiratory illness has necessitated urgent and innovative medical interventions [1].

Among therapeutic options, antiviral therapies are now considered essential in reducing the morbidity and mortality of COVID-19 disease. These therapies target various stages of the viral life cycle, including entry into host cells, replication, and assembly of new viral particles. The antiviral drugs remdesivir and Paxlovid (nirmatrelvir plus ritonavir combination) have been identified as important agents against the virus, each exhibiting some effectiveness in COVID-19 patient management [2].

Remdesivir was granted emergency use authorization (EUA) by the U.S. Food and Drug Administration (FDA) in May 2020, and it was subsequently fully approved for the treatment of COVID-19 among mild to moderately affected hospitalized patients [3,4]. Remdesivir is a nucleotide analogue prodrug that inhibits viral replication by restraining RNA-dependent RNA polymerase (RdRp) of SARS-CoV-2 [5]. Administration of remdesivir may help reduce disease duration and decrease mortality rates among patients admitted with acute illnesses attributable to the COVID-19 pandemic, according to findings from clinical trials [6]. However, its efficacy depends on the timing of administration and other patient-centered interventions in individuals receiving medical care for severe cases of COVID-19.

In December 2021, the FDA gave Emergency Use Authorization to Paxlovid, a new oral antiviral combining ritonavir and nirmatrelvir, a SARS-CoV-2 main protease inhibitor designed to increase the drug’s concentration in blood plasma by boosting pharmacokinetics [7]. This allowed it to be used for emergency treatments in case of a mild or moderate infection due to high-risk factor conditions [8]. Clinical trials conducted with Paxlovid have demonstrated a decreased likelihood of hospitalization or death.

A comprehensive understanding of the mechanisms, efficacy and safety profiles is required for incorporating antiviral therapies such as remdesivir and Paxlovid into clinical practice. Personalized medicine is essential in optimizing the use of these antivirals, which tailor medical treatments to individual patients. Some important factors include patient age, comorbidities, genetic predispositions, and timing of administration of the anti-viral drugs for those patients that will gain maximum benefits from them. Specifically, this method is vital in high-risk groups, which include elderly people, individuals who have other illnesses, and those whose immunity systems are frail.

Further, using remdesivir and Paxlovid in combination or sequentially in certain situations may yield augmented benefits that could improve antiviral efficacy and reduce severe outcomes [9]. Nevertheless, these strategies should be thoroughly investigated through randomized controlled trials and real-world studies to confirm their safety and effectiveness.

Twigging the mechanisms of immune dysregulation and the inflammatory response in COVID-19 is imperative for formulating effective therapeutic strategies. Numerous pharmacological agents, whether used singly or in combination, have undergone and are currently undergoing rigorous testing in clinical trials. However, none of these drugs have shown unambiguous clinical benefits sufficient to be designated as the standard of care. This underscores the need for continued research and innovation in identifying and validating treatments that can reliably improve patient outcomes in COVID-19 [10].

This article will give an integrative analysis of remdesivir and Paxlovid concerning their effectiveness, safety profiles, and how a personalized approach may benefit high-risk individuals who could be more susceptible to developing severe cases. This article aims to review and synthesize the current evidence on remdesivir and Paxlovid in high-risk COVID-19 patients. We want to determine how best to use these antivirals under personalized medicine by examining pharmacodynamics, clinical trial data, and real-world experiences. Our target is to empower healthcare practitioners with holistic understandings that will enhance patient outcomes and ease the burden on healthcare systems, thereby saving lives.

## 2. Materials and Methods

### 2.1. Study Design

A retrospective was conducted between 1 June 2023 and 31 May 2024. All subjects included in the study were patients admitted to the Clinical Emergency Hospital of Bihor diagnosed with COVID-19 and treated with antiviral medications such as remdesivir or nirmatrelvir/ritonavir.

The study received approval from the Clinical Emergency Hospital of Bihor Ethics Committee (Approval No. 33446/07.10.2020) and adheres to the ethical principles of the World Medical Association’s Declaration of Helsinki (1967). Informed consent was obtained from all subjects involved in the study.

The patients were classified based on disease severity according to the clinical and laboratory criteria in Table 1 [11].

**Figure 1:**
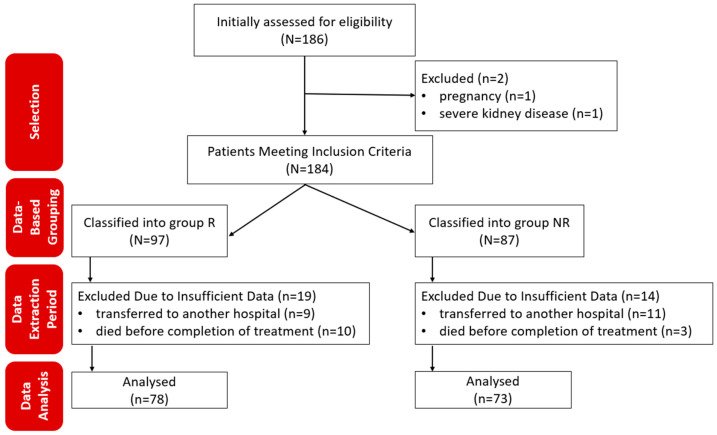
Flowchart of patient inclusion and data analysis in the study. R—remdesivir; NR—nirmatrelvir/ritonavir; *n*—number.

The patients included in the study were selected based on pre-established eligibility criteria, and their clinical data were analyzed considering the inclusion/exclusion criteria without active allocation interventions, Figure 1.

### 2.2. Inclusion Criteria

- COVID-19’s stage I or II;
- Prodrome less than 5 days;
- High-risk factors for progression to severe COVID-19 (age ≥ 50 years, BMI ≥ 25, cancer, chronic kidney/liver/lung diseases, diabetes, heart diseases, other infectious diseases, neurological diseases, immunosuppression, mental diseases, sedentary lifestyle, substance abuse (drugs, alcohol, smoker), disabilities).

### 2.3. Exclusion Criteria

- Severe kidney/liver disease;
- Pregnancy;
- Age < 18 years;
- COVID-19’s stage III;
- Died before the completion of antiviral treatment.

The patients received treatment with one of the two products. The administration of the two medications was as follows:

- Remdesivir—day 1 loading dose of 200 mg intravenously (IV), then from day 2 onwards, 100 mg IV once daily—for 5 days.
- Nirmatrelvir/Ritonavir—300 mg nirmatrelvir plus 100 mg ritonavir taken orally, twice daily, for 5 days.

**Figure 2:**
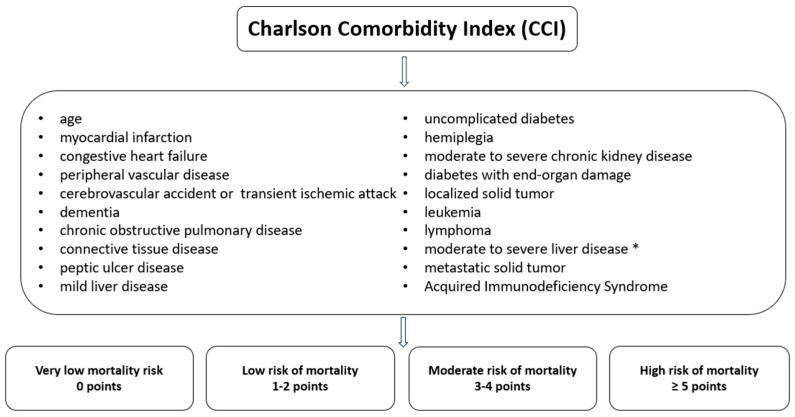
Charlson Comorbidity Index Organizational Chart. * Severe = cirrhosis and portal hypertension with variceal bleeding history; moderate = cirrhosis and portal hypertension but no variceal bleeding history.

The Charlson Comorbidity Index (CCI) is used in clinical medicine and research to assess the severity and impact of comorbidities (19 coexisting conditions) on patients’ prognosis. The CCI was applied to all patients at admission and at the end of antiviral therapy. Each of the 19 conditions included in the CCI is evaluated based on its presence and severity, with a score assigned between 1 and 6 points. These points are summed to obtain the patient’s total score (Figure 2) [12].

### 2.4. Data Collection

Demographic information such as age, sex, and domicile, along with the medical history of previous conditions, clinical details (oxygen saturation (SpO_2_), body mass index (BMI), period from onset to hospitalization (POH), hospitalization period (HP)), and laboratory investigations (complete blood count (CBC), ferritin, D-dimers, C-reactive protein (CRP), lactate dehydrogenase (LDH)), as well as chest computed tomography (CT) scans, were extracted from the patients’ observation records.

Laboratory investigations for LDH, ferritin, and lymphocyte count were evaluated at two time points:

t_0_: Before the start of antiviral treatment;

t_1_: At the end of antiviral treatment.

Oxygen saturation (SpO_2_) was monitored for 5 days in both groups. The patients’ SpO_2_ was measured using a pulse oximeter (Human Accurate Bio-Medical Technology Co., Ltd., Changsha, China).

Partial pressure of oxygen (PaO_2_) was determined from arterial blood through arterial blood gas analysis. The fraction of inspired oxygen (FiO_2_) was calculated based on standard clinical protocols, considering the oxygen flow rate and the method of oxygen administration relative to ambient air. The PaO_2_/FiO_2_ ratio was calculated for each individual patient.

According to World Health Organization (WHO) recommendations, SARS-CoV-2 infection was confirmed either by the detection of SARS-CoV-2 antigens (rapid test) or by a positive real-time polymerase chain reaction (RT-PCR) test. Pharyngeal and oropharyngeal samples were collected and properly transported following WHO guidelines [13].

Laboratory investigations (CBC, CRP, D-dimers, ferritin, and LDH) were collected from venous blood samples using specific vacutainer tubes for each test and processed in the Bihor County Emergency Clinical Hospital laboratory.

The CBC was collected in vacutainer tubes containing ethylenediaminetetraacetic acid (EDTA) as an anticoagulant and was analyzed using the Beckman Coulter 628134 UniCel DxH 800 Hematology Analyzer from Beckman Coulter International S.A., Nyon, Switzerland. The reference range for the tracked cells is as follows:

- Leukocytes: 4.00–10.00 × 10^3^/µL
- Neutrophils: 2.4–6.5 × 10^3^/µL
- Lymphocytes: 1.00–4.00 × 10^3^/µL

To determine D-dimer levels, venous blood samples were collected in vacutainer tubes with 0.105 M sodium citrate and analyzed using the ALINITY AC analyzer (Abbott Laboratories, Abbott Park, IL, USA) by the latex agglutination method with photometric detection. The reference values are <0.5 µg/mL.

Ferritin was collected from venous blood using vacutainer tubes without anticoagulant agents and analyzed using the Alinity Abbott analyzer (Abbott Laboratories, Abbott Park, IL, USA) by the immunochemical method with electrochemiluminescent detection (ECLIA). The reference values, according to age and sex, are as follows:

- Males (>18 years): 30–400 ng/mL
- Females (>18 years): 13–150 ng/mL

PCR values were determined from venous blood samples collected in vacutainer tubes without anticoagulant. The determination was performed using the Beckman Coulter AU5811 analyzer by the estimated MDRD/ALINIQ calculation method. The reference values are 0–5.0 mg/L.

All samples were collected after fasting and were immediately transported to the hospital laboratory for analysis.

### 2.5. Statistical Analysis

The statistical analysis was performed using the Statistical Package for the Social Sciences (SPSS), version 26 (IBM Corp., Armonk, NY, USA). The initial data were presented as numerical value (*n*), proportion (%), mean (M), or standard deviation (SD). The Shapiro–Wilk test was used to assess the normality of data distribution. Statistical significance, represented by the *p*-value, was calculated using the chi-square test, Student’s *t*-test/Wilcoxon signed-rank test for paired data/Mann–Whitney U for independent data, Friedman test for related data sets and the log-rank test for the Kaplan–Meier method. The variable variation for different monitored laboratory parameters was analyzed using sensitivity to change and evaluated by effect size (r) using Z-score, using the formula: r = Z/\sqrt N. Z was the “Z score” (Wilcoxon test), and N was the total number of observations. The r was considered small if r < 0.3, medium for a value between 0.3 and 0.5, and high for r > 0.5. The results were interpreted by correlating the r-value with the *p*-value. A *p*-value < 0.05 was considered statistically significant.

## 3. Results

In total, 184 COVID-19 patients were initially included in the study during the observation period. Of these, 33 patients were later excluded (20 were transferred to another hospital and could not be subsequently monitored, and 13 died before completing the antiviral treatment). A total of 151 subjects completed the study, with 78 patients in the R group and 73 in the NR group. The demographic characteristics of the two groups did not show statistically significant differences (Table 2).

The Shapiro–Wilk normality test indicated a *p* < 0.05 for the tracked data, concluding that the normal distribution of the tracked parameter values cannot be supported, which is why nonparametric tests will be applied.

The differences between the SpO_2_ values over the five days were evaluated using nonparametric tests. The Friedman test revealed significant differences between the SpO_2_ values in the two groups (*p* < 0.001) (Table 3).

**Figure 3:**
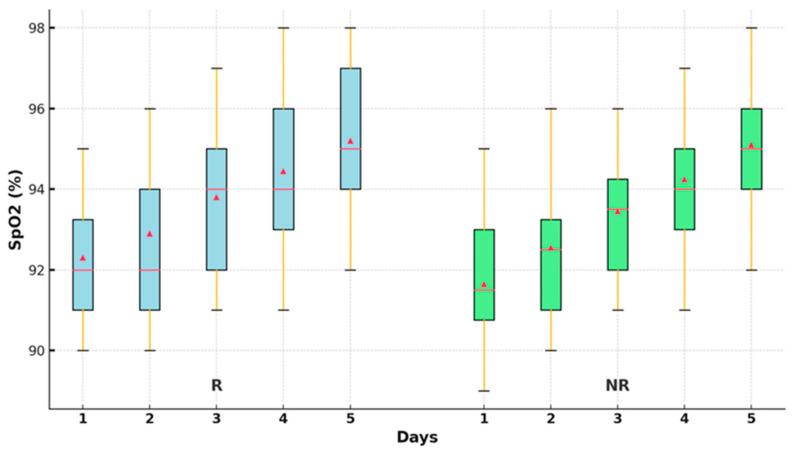
Oxygen saturation values in groups R and NR. SpO2—oxygen saturation levels; R—remdesivir; NR—nirmatrelvir/ritonavir; −—median; ▲—mean.

The oxygen saturation values improved significantly statistically after 48 h of treatment in the R group (*p* < 0.001), compared to the NR group, where a significant increase was observed only in the first 24 h (*p* = 0.010). After that, no statistically significant improvements in the parameter were recorded compared to the first day. Oxygen saturation was statistically improved at the end of the 5-day treatment compared to the time of admission, only in the group R (95.11 ± 1.80; 91.76 ± 1.80; *p* < 0.001) (Figure 3).

**Figure 4:**
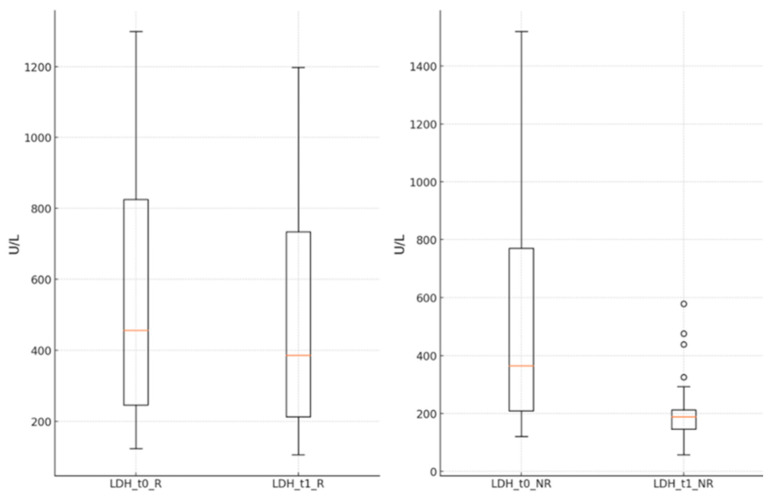
LDH level in groups R and NR, at times t_0_ and t_1_. LDH—lactate dehydrogenase; t_0_—starting time of the antiviral; t_1_—end time of the antiviral; R—remdesivir; NR—nirmatrelvir/ritonavir.

The LDH values significantly decreased in both groups at the end of the antiviral treatment compared to the time of hospital admission (R group: 572.33 ± 355.03 U/L vs. 487.99 ± 304.76 U/L, *p* < 0.001; NR group: 506.51 ± 371.62 U/L vs. 195.49 ± 79.78 U/L, *p* < 0.001) (Figure 4).

**Figure 5:**
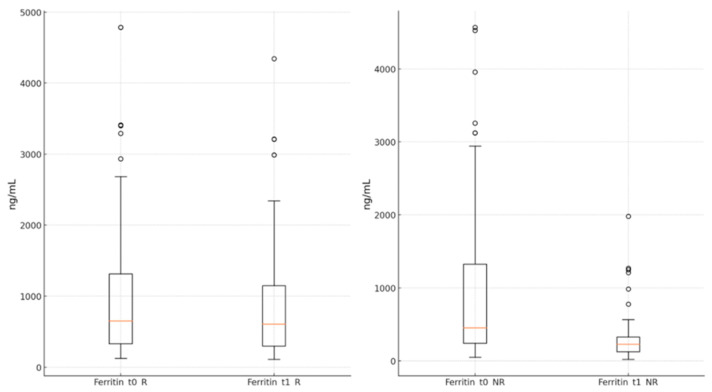
Ferritin level in groups R and NR, at times t_0_ and t_1_. t_0_—starting time of the antiviral; t_1_—end time of the antiviral; R—remdesivir; NR—nirmatrelvir/ritonavir.

The ferritin levels showed a significant reduction in both groups by the end of the antiviral treatment compared to the moment of admission (R group: 894.89 ± 836.15 ng/mL vs. 1040.96 ± 996.93 ng/mL, *p* < 0.001; NR group: 313.97 ± 324.95 ng/mL vs. 956.07 ± 1063.43 ng/mL, *p* < 0.001) (Figure 5).

**Figure 6:**
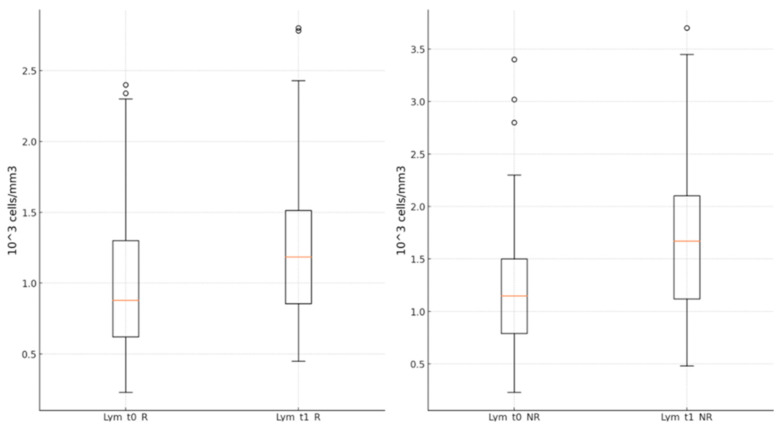
Lymphocyte values in groups R and NR, at times t_0_ and t_1_. t_0_—starting time of the antiviral; t_1_—end time of the antiviral; R—remdesivir; NR—nirma trelvir/ritonavir.

Lymphocyte values were statistically significantly higher in both groups at the end of the antiviral treatment than upon hospital admission (R group: 1.23 ± 0.51 (×10^3^ cells/mm^3^) vs. 0.99 ± 0.49 (×10^3^ cells/mm^3^), *p* < 0.001; NR group: 1.63 ± 0.69 (×10^3^ cells/mm^3^) vs. 1.16 ± 0.62 (×10^3^ cells/mm^3^), *p* < 0.001) (Figure 6).

The statistical analysis performed using the Wilcoxon test for variations in LDH, ferritin, and lymphocyte count in the R and NR groups at the beginning and end of antiviral treatment indicated a statistically significant variation in all groups (*p* < 0.001). The effect size calculated with the r coefficient indicates a significant change in LDH values in the R group (r = 0.86, Z = −7.579, *p* < 0.001), with a slightly smaller but still significant effect in the NR group (r = 0.84, Z = −7.183, *p* < 0.001). A significant change was recorded for ferritin in both the R and NR groups (r = 0.87, Z = −7.673, *p* < 0.001; r = 0.87, Z = −7.425, *p* < 0.001). For lymphocytes, a moderate but statistically significant effect was identified in the R group (r = 0.53, Z = −4.639, *p* < 0.001), and a large effect in the NR group (r = 0.87, Z = −7.425, *p* < 0.001).

**Figure 7:**
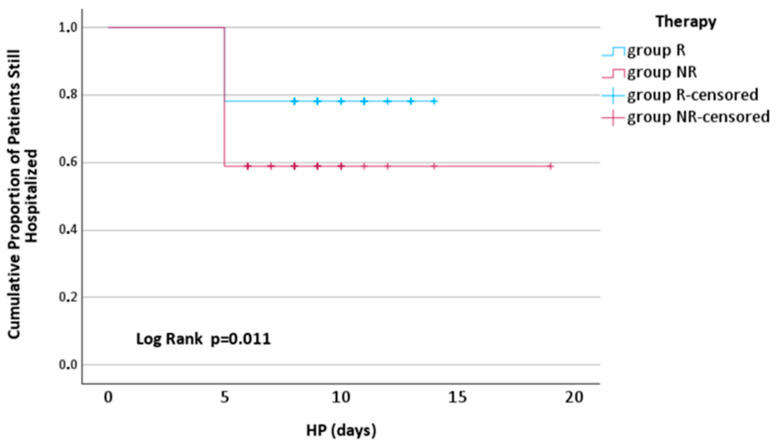
Kaplan–Meier curves for time to discharge by therapy. HP—the hospitalization period; R—remdesivir; NR—nirmatrelvir/ritonavir.

At 5 days after admission, significantly fewer patients from group R were discharged compared to those from group NR (17 (21.79%) vs. 30 (41.09%), *p* = 0.011) (Figure 7).

The mortality rate was significantly higher in patients from group R compared to group NR (23 (29.48%) vs. 5 (6.84%), *p* < 0.001). No statistically significant differences were recorded in the number of days from admission to the death of patients in the R group compared to the NR group (10.09 ± 1.76 vs. 9.25 ± 3.69, *p* = 0.684).

The patients in the R group experienced more adverse reactions than those in the NR group (Table 4).

## 4. Discussion

There has been an emphasis on finding ways to treat COVID-19 so that complications or deaths could be minimized. The two antiviral drugs that are causing much interest include remdesivir and Paxlovid. Both are used to tackle COVID-19 in different ways based on the virus’s progression and the patient’s condition.

COVID-19 primarily impacts the respiratory system, leading to inflammation and lung damage, which hampers lung efficiency in oxygenating the blood. Oxygen level reduction indicates respiratory failure, even if the patient has not presented severe symptoms [13]. In some patients with COVID-19, the so-called ‘silent hypoxemia’ is where oxygen saturation is decreased, and the patient does not complain of shortness of breath. Observing the patient’s saturation level can detect such situations before the patient’s condition worsens sharply. If the oxygen saturation level drops below 93%, severe health implications could call the need for critical acts of medical necessity. In the current study, oxygen saturation levels were significantly better 48 h after commencing remdesivir therapy compared to the nirmatrelvir/ritonavir treated group, in which oxygen saturation was improved at 24 h, but, after, it remained unchanged. This was also observed in the clinical trial by Aye T. et al., where the treated group comprised 204 patients who were administered remdesivir. It showed that using remdesivir can shorten the recovery time of patients with pneumonia on supplemental oxygen if started during the first 4 days of onset of symptoms [14]. The same study also noted that the patients were given this treatment regardless of the severity of SARS-CoV-2 symptoms, whether the patient was having hypoxia, or if hospitalization was required, and if not, as a criterion for administering remdesivir. Regarding the effect of Paxlovid treatment on oxygen saturation, there is evidence that the drug lowers the likelihood of progressing into severe disease forms, including respiratory failure, associated with decreased oxygen saturation. This study also found that patients with Paxlovid are at a significantly lower risk of being hospitalized or requiring mechanical ventilation than those who did not [15]. Thus, there are no specific scientific works that reveal the increase in oxygen saturation as a direct effect of Paxlovid, but the decrease in the transition to severe forms of the disease and the prevention of lung damage means maintaining adequate oxygen saturation.

Inflammation is highly relevant in COVID-19 disease progression and the effects of potential treatments regarding severity and overall outcome. C-reactive protein (CRP), ferritin, D-dimers, and LDH are used to assess systemic inflammation and can be informative about antiviral therapies. The present study shows that LDH, ferritin, and lymphocyte levels improved noticeably after remdesivir treatment compared to nirmatrelvir/ritonavir, where the difference was not as prominent. Singh S. and colleagues described the impact of remdesivir treatment that resulted in the decreased lymphocyte count, while the levels of CRP, ferritin, and LDH were not affected [16], compared to the present study where substantial changes in inflammatory markers were observed after remdesivir treatment. This has been linked to decreased inflammation, which relates to improvement, as manifested by reduced oxygen requirements and shorter hospital stays. The decrease in the values of inflammatory markers in the Paxlovid-treated group was also recorded. Hammond J. and colleagues also worked on the effects of the Paxlovid drug, which can affect the extent of inflammation of patients based on the level of CRP. Patients who took Paxlovid had lower levels of CRP than those who did not take the treatment. This implies that Paxlovid could help decrease systemic inflammation response in COVID-19 patients to avoid clinical worsening [17]. Pfizer conducted the trial on Paxlovid in the phase 2/3 portion of the EPIC-HR study. While LDH was not highlighted as a key variable for the definitive analysis of primary reports of the study, patients treated with Paxlovid had lower rates of progression to severe forms of the disease and hospitalizations. LDH levels were measured in some patients while assessing the degree of tissue injury and inflammation; however, the study did not indicate information on the changes in LDH levels [18]. Although there are no definitive risks and interactions between Paxlovid and LDH in published research, patient data imply that the treatment will lower LDH by reducing viral activity and inflammation and preventing cell damage. Regarding the effects on LDH and other inflammatory markers, this subject shall be further explored in detail by future research. As far as we know, no comparative clinical studies between the two pharmaceutical products address the progression of laboratory parameters in COVID-19 patients.

Shortening the hospital stay period is advantageous from different viewpoints for the patient and healthcare organizations. For example, if a patient is admitted to a hospital and must stay there long, the chances of developing nosocomial infections increase. Being admitted incurs a lot of expenses for the patient and the healthcare system at large. Less hospitalization brings down direct expenses on medical treatment, hospital expenses, medical care decreases and other eventual expenses due to loss of working days. This leads to better cost-efficiency in terms of financial and manpower resources. Fewer patients occupying hospital beds translates to more resources available to other patients who need the hospital’s services. In the case of a pandemic or, for instance, during periods of high patient influx, a great demand is put on the number of available beds; therefore, discharging patients more quickly is more advantageous for a hospital. Fewer hospital stays decrease the concentration of doctors and nurses, thus increasing their capacity to treat many patients effectively. Reduced hospital stay also enhances patient’s quality of life in the sense that they can get back to their activities and normal life easily. Especially in elderly patients, bed rest in the hospital can result in higher levels of dependency and cognitive decline, and early mobilization reduces or minimizes such adverse outcomes. Reduced length of stay is important for the patient because it results in faster recovery with fewer complications and is convenient for the healthcare system as it means rationed use of resources and lower expenses. The remdesivir’s efficacy was illustrated in the ACTT-1 study, in which patients’ hospitalization period was shortened when the drug was administered, with an average hospital stay of 11 days [19]. The findings of the present investigation revealed a lower number of patients from the remdesivir group being discharged than the Paxlovid group. The EPIC-HR trial showed that Paxlovid lowers the risk of hospital admission among patients with mild to moderate COVID-19. The study included patients for whom antiviral therapy was initiated within 5 days of disease onset, resulting in no direct impact on the duration of hospitalization [18]. Studies on remdesivir and Paxlovid therapy primarily evaluated the impact of each treatment on COVID-19 progression and severity, including the duration of hospitalization for each drug, with no studies comparing them directly.

The mortality index in COVID-19 patients treated with remdesivir has been investigated in numerous clinical trials, and its outcomes differ depending on the sample and the severity of the cases. However, in this study, the mortality rate was higher in the remdesivir group than in the nirmatrelvir/ritonavir group. In a randomized controlled trial sponsored by the NIAID in the U.S., remdesivir was surprisingly found to reduce the recovery time of COVID-19 patients but failed to statistically reduce the deaths at 29 days (11.4% among patients treated with remdesivir vs. 15.2% among patients in the placebo group) [19]. Studies by Amirizadeh M. and colleagues also identified no advantage of remdesivir in reducing mortality [20]. The SOLIDARITY study was led by WHO and aimed to assess the efficacy of several treatments for COVID-19, one of which is remdesivir. Unfortunately, the study did not show a decrease in the mortality rate among patients who took remdesivir compared with other standard treatments, and it stood at 11.9% in the remdesivir group and 11% in the placebo group [21]. Remdesivir has been proven to reduce the recovery time of COVID-19 patients. Its impact on mortality is limited and can vary depending on factors such as the severity of the illness and when treatment begins. Ritonavir has become a recognized treatment for COVID-19, especially for those at high risk of developing severe symptoms. Clinical trials and research studies have provided information on how Paxlovid affects the mortality rates in individuals with COVID-19. The study called EPIC HR (Evaluation of Protease Inhibition, for COVID-19 in High-Risk Patients), which was randomized and double-blind to evaluate the effectiveness of Paxlovid in patients at risk of outcomes who are not hospitalized, has shown that starting Paxlovid treatment within three days of symptoms can reduce the risk of hospitalization or death by about 89% compared to those who received a placebo. It is interesting to note that no deaths were reported among the group taking Paxlovid (out of 1039 people), while the placebo group had 12 fatalities (a rate of 1%) [18]. Furthermore, Zong K’s retrospective study, with 1018 patients, showed that those who received Paxlovid had mortality rates after 28 days compared to the population [17]. In a study conducted by Rajme-López S. et al. in Mexico City in 2022–23, involving a cohort of 783 high-risk outpatients with COVID-19 (451 treated with remdesivir and 332 with nirmatrelvir/ritonavir), of whom 97.8% had at least one comorbidity, a significant reduction in the risk of death was observed in patients treated with nirmatrelvir/ritonavir at 28 days [22]. This study’s results support the present study’s findings, in which the mortality rate was higher in the group treated with remdesivir. On the other hand, in a study conducted by Basouliu D. et al., involving a cohort of 521 high-risk COVID-19 patients treated with either 3-day remdesivir (356 patients) or nirmatrelvir/ritonavir (165 patients), the risk of severe COVID-19 progression and mortality did not differ significantly between the two groups [23]. The medical literature remains contradictory regarding the mortality rates of the two therapeutic products.

In the present study, patients treated with remdesivir were the group that frequently experienced adverse effects compared to the group treated with nirmatrelvir/ritonavir. The most common adverse effects included nausea, skin rashes, headaches, and liver impairment. In the ACTT-1 study, the most common adverse effects reported in patients treated with remdesivir were nausea, headache, and elevated liver enzyme levels (transaminases), indicating the possibility of liver damage [19]. In the EPIC-HR study, the most common adverse reactions in patients treated with Paxlovid included taste disorders (dysgeusia), diarrhea, headaches, and nausea. Dysgeusia, or a metallic taste in the mouth, was reported by many patients [18]. Careful monitoring of patients and proper management of drug interactions are essential for the safe use of these treatments.

Antiviral therapy for COVID-19 has evolved significantly with the introduction of remdesivir and Paxlovid, providing clinicians with more varied and adaptable options for managing this complex disease. However, the effective use of these drugs depends on a deep understanding of the patient’s profile and the judicious application of updated clinical guidelines. A personalized approach to patient care considers specific risk factors such as age, comorbidities, or immunosuppression, involving a therapeutic management strategy that differs from the standard approach, which may not emphasize these aspects.

### Strengths and Limitations

The study presents a comparative analysis between remdesivir and Paxlovid in COVID-19 treatment, a perspective rarely explored in medical research. It highlights unique aspects, some of which are addressed for the first time in the literature, thereby contributing to the optimization of personalized treatment for high-risk patients. This retrospective study presents certain specific limitations. The use of previously collected data may limit the ability to control for certain relevant variables, and it may contain incomplete information, impacting the precision of the final interpretation of the results. Patient selection and the administered antiviral treatment varied based on regional criteria and the individual decisions of medical teams. Considering these observations, future prospective and controlled studies are necessary to confirm the conclusions presented in this study.

## 5. Conclusions

The current study suggests that remdesivir might be one of the most promising options for the treatment of hospitalized adults with SARS-CoV-2 infection associated with comorbidities since, so far, it has yielded positive results in terms of reducing risks of serious complications, keeping inflammation under control, and ensuring good tolerability. However, no significant reduction in mortality was observed. On the other hand, in the Paxlovid group, lymphocyte count exhibited a very noticeable negative impact, with a decrease between the first and last values in that group. Moreover, hospitalization time in the Paxlovid-treatment group was shorter compared to the remdesivir treatment group.

COVID-19 can cause a host of short-term and long-term complications; thus, the sooner antiviral therapy is initiated from the beginning of the illness, the better. Further studies are indicated to better identify the population for whom each of these treatments would offer maximum benefit and to modify treatment based on the emergence of new SARS-CoV-2 variants and the changing pandemic landscape.

## Figures and Tables

**Table 1:** Characteristics of COVID-19’s stage.

COVID-19’s Stage	Clinical Manifestations	Laboratory or Radiological Abnormalities
I (mild)	mild constitutional symptoms, dry cough, headache, fever	lymphopenia
II (moderate)	cough, high fever, dyspnea	lymphopenia, elevated transaminases, increased but not remarkable levels of inflammatory markers, normal serum procalcitonin level, abnormal thoracic imaging
a	without hypoxemia (PaO_2_/FiO_2_ > 300 mm Hg)	
b	with hypoxemia (PaO_2_/FiO_2_ < 300 mm Hg)	
III (severe)	acute respiratory distress syndrome, sock, cardiac failure, severe respiratory failure or systemic organ involvement	significantly high levels of C-reactive protein, ferritin, D-dimer, abnormal thoracic imaging

PaO_2_/FiO_2_—partial pressure of oxygen/fraction of inspired oxygen.

**Table 2:** Characteristics of COVID-19 groups.

Parameter	R Group (78)	NR Group (73)	*p*-Value
DD			
Age, years, M ± SD	63.54 ± 13.12	66.27 ± 16.38	0.258 ^a^
Male gender, *n* (%)	42 (53.85)	33(45.218)	0.298 ^b^
Urban residence, *n* (%)	35 (77.42)	44 (56.83)	0.311 ^b^
SpO_2_, M ± SD	91.76 ± 1.57	92.30 ± 2.22	0.062 ^c^
Without hypoxia, *n* (%)	29 (37.17)	36 (49.31)	0.385 ^b^
POH, M ± SD	3.14 ± 0.97	3.19 ± 0.98	0.751 ^a^
PMH			
BMI, M ± SD	28.63 ± 4.18	27.52± 3.36	0.075 ^a^
CCI, M ± SD	4.91 ± 2.07	4.38 ± 2.12	0.125 ^a^
Paraclinical investigations, M ± SD			
WBC, (×10^3^/mm^3^)	11.50 ± 3.91	10.17 ± 4.94	0.067 ^c^
Lym, (×10^3^/mm^3^)	0.99 ± 0.449	1.16 ± 0.62	0.082 ^c^
No, (×10^3^/mm^3^)	7.87 ± 3.83	6.71 ± 3.75	0.062 ^c^
CRP, (mg/dL)	151.35 ± 95.62	119.67 ± 93.90	0.054 ^c^
Ferritin, (ng/mL)	1040.96 ± 996.93	956.07 ± 1063.43	0.078 ^c^
D-dimer, (ng/mL)	988.32 ± 1033.34	681.89 ± 1097.095	0.080 ^c^
LDH, (U/L)	572.33 ± 355.03	506.50 ± 371.62	0.124 ^c^
COVID-19’s stage at admission, *n* (%)			
Stage I	10 (12.82)	12 (16.44)	0.669 ^b^
Stage II a	19 (24.36)	24 (32.88)	0.445 ^b^
Stage II b	49 (62.82)	37 (50.68)	0.195 ^b^

DD—demographic data; SpO_2_—oxygen saturation levels; PMH—past medical history; POH—period from onset to hospitalization; BMI—body mass index; CCI—Charlson Comorbidity Index; WBC –white blood cells; Lym—lymphocytes; No—neutrophils; CRP—C-reactive protein; LDH—lactate dehydrogenase; M—mean; SD—standard deviation; R—remdesivir; NR—nirmatrelvir/ritonavir; *n*—number; *p*-value as determined by ^a^ *t*-test, ^b^ Chi-square test, and ^c^ Mann–Whitney U.

**Table 3:** The differences between the SpO_2_ values.

Test	Parameter	Mean Rank	Chi-Square (χ²)	df	*p*-Value
Friedman Test	SpO_2__R_1	1.70	217.025	4	<0.001
	SpO_2__R_2	1.91			
	SpO_2__R_3	2.99			
	SpO_2__R_4	3.65			
	SpO_2__R_5	4.76			
	SpO_2__NR_1	2.80	30.913	4	<0.001
	SpO_2__NR_2	2.36			
	SpO_2__NR_3	2.99			
	SpO_2__NR_4	3.25			
	SpO_2__NR_5	3.60			

SpO_2_—oxygen saturation levels; R—remdesivir; NR—nirmatrelvir/ritonavir.

**Table 4:** Adverse effects for groups.

Parameter	Group R (78)	Group NR (73)	*p*-Value
Adverse effects			
Nausea, *n* (%)	21 (16.38)	16 (11.68)	0.411 ^a^
Skin rash, *n* (%)	3 (2.34)	2 (1.46)	0.654 ^a^
Headache, *n* (%)	3 (2.34)	0 (0)	0.083 ^a^
Hepatocellular injury syndrome, *n* (%)	12 (9.36)	0 (0)	<0.001 ^a^
Total, *n* (%)	39 (30.42)	18 (13.14)	0.005

*n*—number; R—remdesivir; NR—nirmatrelvir/ritonavir; *p*-value as determined by ^a^ *t*-test.

## Data Availability

The data are not publicly available due to it being a part of an ongoing doctoral study.
